# Resting EEG, Hair Cortisol and Cognitive Performance in Healthy Older People with Different Perceived Socioeconomic Status

**DOI:** 10.3390/brainsci10090635

**Published:** 2020-09-15

**Authors:** Carolina Villada, Mauricio González-López, Herlinda Aguilar-Zavala, Thalía Fernández

**Affiliations:** 1 Departamento de Neurobiología Conductual y Cognitiva, Instituto de Neurobiología, Universidad Nacional Autónoma de México, 076230 Queretaro, Mexico; c.villada@ugto.mx (C.V.); mau_89@comunidad.unam.mx (M.G.-L.); 2 Departamento de Psicología, División de Ciencias de la Salud, Universidad de Guanajuato, 37670 Leon, Mexico; 3 División de Ciencias de la Salud e Ingenierías, Campus Celaya-Salvatierra, Universidad de Guanajuato, 38110 Celaya, Mexico; h.aguilar@ugto.mx

**Keywords:** resting EEG, hair cortisol concentration, cognitive performance, healthy older people, socioeconomic status

## Abstract

Successful aging depends upon several internal and external factors that influence the overall aging process. Objective and subjective socioeconomic status emerge as potential psychosocial factors in the ethiopathophysiology of aging-related disorders. Presumably, low socioeconomic status can act as a psychosocial stressor that can affect humans’ physiology via psychoneuroendocrine mechanisms, that may, in turn, affect the brain physiology. In resting-state electroencephalography (EEG), excess theta and delta activity has been related to cognitive decline and dementia. The main aim of this study was to analyze the effect of objective and subjective socioeconomic status (SES) on cognition and brain electrical activity through EEG measures. The present research constitutes a cross-sectional study with thirty healthy older adults (61–82 years old) separated into two clusters: high socioeconomic (HS) and low socioeconomic (LS) status; they were evaluated and compared in cognitive terms using the Wechsler Adult Intelligence Scale (WAIS-IV). An EEG at rest was recorded to measure brain activity and, as an indicator of long-term stress exposure, hair cortisol concentrations (HCC) were measured. Our results show that lower SES is related to a worse performance in working memory tasks (*p* = 0.009), higher delta (*p* = 0.002) and theta power (*p* = 0.039), and lower alpha activity (*p* = 0.028). However, it seems that SES does not significantly affect HCC in this population of healthy older adults. The effects of SES on long-term cortisol exposure, brain electrical activity, and cognitive functions in healthy older people emphasize the role of psychosocial factors in aging from an integrative perspective that will allow us to implement better prevention programs to target cognitive decline in adults.

## 1. Introduction

It is well known that life expectancy is increasing progressively, a phenomenon that is called population aging. As a consequence, the incidence of neurocognitive disorders, as well as other age-related diseases, is increasing around the world. Hence, it is becoming a challenge for researchers to elucidate the factors involved in the development of cognitive decline.

The assessment of cognitive decline is mostly clinical but, in addition to neuropsychological batteries for evaluating cognition, there are multiple psychophysiological tools that could help us in research programs, such as event-related potentials, brain imaging (functional or structural MRI), and electroencephalography (EEG), among others. EEG, together with contemporary analysis methods, is a reliable tool to differentiate several cognitive disorders, from mild cognitive impairment (MCI) to Alzheimer’s disease (AD) [1]. However, the most important goal in the detection of cognitive decline is to find signs of abnormalities prior to the establishment of cognitive deterioration. In this line, Prichep et al. (2006) [2] found—in a sample of healthy older adults—that the higher the theta power and the lower the mean frequency, the more probable it was to develop cognitive decline after 7–9 years. Moreover, current source analyses of these subjects in the theta band showed that the brain structures responsible for this abnormal activity in the surface EEG were the hippocampus, amygdala, and temporoparietal cortex [3].

Other studies, which assessed the progression from MCI to AD, observed that those who progress towards AD showed higher theta and delta power, lower alpha power as well as an altered frontoparietal coherence [4,5,6]. Cognitive decline has also been associated with higher theta power and lower beta power [7,8]. Studies in healthy elderly subjects reported increases in delta activity and decreases in beta activity and decreases in the mean frequency over a 2.5-year period [9]. Nakano et al. (1992) [10] found that a decrease in alpha activity and a significant increase in theta activity were related to a decline in cognitive function.

In addition, one of the most relevant factors related to the incidence of cognitive decline is prolonged stress exposure. It is well known that cortisol, the end-product of the hypothalamus–pituitary–adrenal (HPA) axis, has a direct effect on some brain structures (i.e., prefrontal cortex and hippocampus) due to the high density of glucocorticoid and mineralocorticoid receptors in these areas [11]. Recently, hair cortisol concentration (HCC) has been considered one biomarker of long-term stress exposure. Due to the fact that hair grows approximately 1 cm/month [12,13], this biomarker can reflect one-month exposure to cortisol for each centimeter of hair collected from the scalp (i.e., 3 cm = 3 months of cortisol exposure). To date there is no consensus regarding the relationship between HCC and cognitive processes. Pulopulos et al. (2014) [14] found that more HCC is related to better working memory in healthy older adults, while others found no relationships between HCC and several measures of cognitive performance in nurses (20–29 years old) [15], and, more recently, negative associations have been reported among HCC, memory and global cognition [16]. These discrepancies could be explained by other factors that can influence cortisol exposure, among them, we can highlight chronic stressors such as socioeconomic status (SES).

Although the relationship between SES and the stress response system has been widely reported [17,18], very few studies have looked into these relationships using HCC as a measure. Recent studies reveal negative relationships among hair cortisol concentrations and SES in children [19]; however, there are few studies about the relationships between SES and HCC in adults. For example, Ursache et al. (2017) [20] found that SES is negatively related to HCC in parents of children with internalizing symptoms, and HCC is also positively related to perceived discrimination in obese adults [21].

In addition, SES can moderate age-related differences in the brain’s functional networks’ organization in middle-age [22]. In older people, higher SES is also associated with a more efficient frontal activity [23] as well, with better white matter integrity [24]. When measuring SES, it is typically conducted using population-specific questionnaires (objective SES). However, it appears that the personal perception regarding SES, that is, the subjective socioeconomic status (SSS), is an even more relevant stress-related factor for cognitive deterioration [25,26,27]. The positive relationship among SSS and health outcomes, such as self-rated health and psychological well-being, has been established in several countries from young adults to elderly individuals [28]. The SSS has also been considered as a predictor of other health outcomes, such as depression, cardiovascular disease and diabetes, among others [29], and, more recently, Zahodne et al. (2018) [30] concluded that, regardless of objective SES, a lower SSS in the elderly can be harmful for their physical, mental, and cognitive health, which is reflected in initial evaluations of memory, but does not predict memory decline. Indeed, it is in older populations where this variable (SSS) seems to have advantages over objective measures of SES as a predictor of health outcomes [31]. These findings highlight the necessity to study both objective SES and SSS in relation to some biomarkers of stress (e.g., hair cortisol) and aging, particularly cognitive aging.

Taking into account the aforementioned, the first objective of this study was to analyze the effect of objective and subjective SES on cognition and brain electrical activity (EEG). We hypothesized that people with lower SES would display worse cognitive performance, which may be related to slower EEG activity. The second objective is to explore if SES affects the HCC in healthy older people as well as the direction of this relationship.

## 2. Materials and Methods

The present research constitutes a cross-sectional study. The entire study was conducted in the Laboratory of Psychophysiology of the Institute of Neurobiology at the National Autonomous University of Mexico, in Juriquilla, Queretaro, Mexico.

### 2.1. Participants

From a total of 49 volunteers, a convenience sample consisting of thirty-one subjects (14 women, 17 men) between 61 and 82 years of age met the inclusion criteria: the subjects had to be active, should have at least 9 years of formal schooling, and their IQ should be greater or equal to 80 measured with the 4th version of the Wechsler Adult Intelligence Scale (WAIS-VI) [32]. None of them had major socioeconomic disadvantages (The Mexican Association of Marketing Research and Public Opinion Agencies; AMAI 8 × 7 questionnaire) [33].

The Mini-Mental State Examination (MMSE) and a structured psychiatric interview (MINI PLUS) were administered to rule out psychiatric disorders. Individuals who exhibited any of the following conditions were excluded: anemia, neurological or psychiatric disorders, uncontrolled hypercholesterolemia, thyroid dysfunction, diabetes, or hypertension. All the post-menopausal women had had their last menstrual period more than 2 years prior to the time of testing, and none of these women had received estrogen replacement therapy. Head trauma with loss of consciousness, history of alcoholism, smoking more than five cigarettes a day, having been under general anesthesia once or more than once in the past year, the presence of a major stressful life event during the last year, and not completing the assessments were also considered exclusion criteria. The main incentive for volunteers was free access to their results of the clinical screening. Subjects signed an informed consent form, as stipulated by the Declaration of Helsinki (2008). The Ethical Committee of the Institute of Neurobiology at the National Autonomous University of Mexico approved this study (reference: 030.H-RM).

### 2.2. Procedure

Participants were recruited via announcements in several mass media and others were derived to our laboratory by participants of previous studies. The data were collected between October 2017 and June 2018. A group meeting took place in the installations of the Institute of Neurobiology. At this meeting (1.5 h), the researchers explained to the future volunteers the main objectives of the study, some characteristics of the variables and techniques involved in the experiments, and addressed possible doubts regarding the study. After this, the participants were scheduled to attend their first session, which consisted of a screening interview to rule out psychiatric and neurological disorders. This was assessed using standardized tools (MINI PLUS and MMSE). In this session, other sociodemographics were gathered, such as educational level and objective and subjective SES, among others. In the second session, the EEG at rest was recorded in the eyes-closed condition by a single qualified technician. Participants were asked to follow some recommendations prior to this second session: (i) attend with clean and dry head, shampoo only, avoid conditioner and other cosmetic products, (ii) no makeup, (iii) avoid consumption of alcohol, stimulants or drugs the day before and on the day of the session, (iv) do not stop the medication they were taking, except for medical prescription, (vi) do not fast, (vii) if possible, use cotton clothing, and (viii) maintain their general habits.

**Figure 1:**
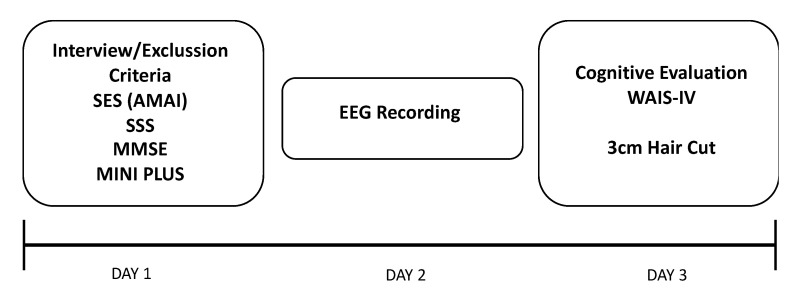
Schematic representation of the experimental procedure. SES: socioeconomic status; AMAI: questionnaire of the Mexican Association of Marketing Research and Public Opinion Agencies; SSS: subjective socioeconomic status; MMSE: Minimental State Examination; MINI PLUS: Mini International Neuropsychiatric; WAIS-IV: 4th version of the Wechsler Adult Intelligence Scale.

The third session consisted of a cognitive evaluation using WAIS-IV. This evaluation was always performed by the same experimenter, who also cut 3 cm hair samples at the end of this session (see Figure 1). The three sessions were carried out between 10.00 am and 2.00 pm. The three sessions were not necessarily scheduled for consecutive days; however, the time between them did not exceed two weeks.

### 2.3. Outcome Measures

All participants underwent a series of assessments on four different domains: socioeconomic, cognitive, electroencephalographic, and endocrine.

#### 2.3.1. Objective Socioeconomic Status AMAI

To measure objective SES, we used the socioeconomic level questionnaire provided by the Mexican Association of Marketing Research and Public Opinion Agencies (AMAI 8 × 7, 2016) [33]. The AMAI questionnaire is based on a statistical model that allows to classify Mexican households in seven levels, according to their capacity of meeting the needs of all members of the household (i.e., the head of household’s education level, number of rooms, number of cars, etc.). The data are classified into an ordinal level according to the scores, which range from 0 to 205 or more. 

#### 2.3.2. Subjective Socioeconomic Status

To measure the SSS, we used the visual analogue scale described by Adler et al. (2000) [34]. To fill this scale, participants were given a drawing of a ladder with 10 rungs. The participants received the following instruction: “Think of this ladder as representing where people stand in our society. At the top of the ladder are the people who are the best off, those who have the most money, most education, and best jobs. At the bottom are the people who are the worst off, those who have the least money, least education, and worst jobs or no job.” They were then asked to place an X on the rung that best represents where they think they stand on the ladder.

#### 2.3.3. Cognitive Evaluation

WAIS-IV, the Wechsler Adults Intelligence Scale [32], is a clinical instrument that evaluates the intelligence of adults from 16 to 89 years old individually. The WAIS-IV provides composite scores, which reflect the intellectual function in four cognitive indices (verbal comprehension, perceptual organization, working memory, and processing speed) and a composed score that reflects the general intellectual quotient (Total IQ). The Spanish version had a Cronbach’s alpha ranging from α = 0.75 to α = 0.91.

#### 2.3.4. Electroencephalogram (EEG)

The EEG recording is a non-invasive technique. EEG represents the brain electrical activity measured by means of sensors placed on the scalp of an individual. It depicts the voltage changes along time.

##### EEG Recording

Subjects were seated in a comfortable chair in a dimly-lit room. The EEG was recorded at rest with eyes closed from 19 channels (10–20 International System) using linked earlobes as a reference. All electrode impedances were at or below 10 kΩ. The amplifier bandpass filter was set from 0.5 to 50 Hz. The EEG was sampled every 5 milliseconds using a MEDICID^TM^ IV System (*Neuronic Mexicana*, *S*.*A*.; Ciudad de México, México), with a gain of 20,000. It was ensured that the participants were in wakefulness at rest condition; in the event that the frequency or amplitude of the posterior alpha rhythm was reduced during a recording, a pause was made to increase the participant’s level of wakefulness. One participant was excluded because he reported to be meditating during the recording.

##### EEG Edition and Analysis

Edition and analyses were carried out off-line. An expert electroencephalographer, using visual inspection, selected twenty-four artifact-free segments of 2.56 s for quantitative analysis. The cross-spectral matrices were calculated using a fast Fourier transform with a frequency resolution of 0.39 Hz, and the following measurements were obtained from each referential lead: the absolute (AP) and relative (RP) powers in each of four frequency bands: delta (1.5–3.5 Hz), theta (3.6–7.5 Hz), alpha (7.6–12.5 Hz), and beta (12.6–19 Hz). A geometric power correction, which has been reported to account for 42% of the variability that is not related to physiological factors [35], was applied. A normative database provided by MEDICID IV [36] was used to calculate z values as follows:

(1) $$Z=\left(x-\bar{x}\right)/S$$

where x is the value for a particular subject, and $\bar{x}$ and S are the mean value and the standard deviation, respectively, of the normative sample considering the age of the subject.

#### 2.3.5. Hair Cortisol Concentration

Hair strands were cut as close as possible to the scalp from a posterior-to-vertex position. Based on an average hair growth rate of 1 cm per month [37], each 3 cm hair segment reflects hair growth for an approximate period of 3 months. For the analysis, each hair segment was washed three times for 3 min with 2.5 mL isopropanol in a 15 mL Falcon tube on a horizontal tube holder for vortex. It was then allowed to dry for at least 48 h under a clean and protected hood; when hair was completely dry, hair segments were transferred into a mortar, and they were completely disrupted. An amount of 15 to 40 mg of powdered hair was transferred into a 2 mL cryovial (Eppendorf of Thermo Fisher Scientific Mexico, Ciudad de Mexico, Mexico). For cortisol extraction, 1.5 mL of pure methanol was added. The vials were then slowly rotated over a period of 24 h after centrifugation in a microcentrifuge (14,000 rpm for 6 min), and the clear supernatant was transferred into a new 2 mL cryovial to let the alcohol evaporate using a centrifugal concentrator Centri Vap (Labconco of Labconco Corporation, Kansas City, MO, USA). When methanol was completely evaporated off, and only the cortisol was left in the tube, the next step was to reconstitute the sample adding a neutral buffer (deionized water). For all samples, cortisol determination was carried out using a commercially available enzyme immunoassay kit for the quantitative in vitro measurement of active free cortisol in saliva (LDN^®^, Northorm, Germany). The intra-assay and inter-assay coefficients of variance for this assay were 4.8% and 6.3%, respectively. The analytical sensitivity of the assay was 0.024 ng/mL, and the range of the assay was between 0.1–30 ng/mL.

### 2.4. Statistical Analysis

**Figure 2:**
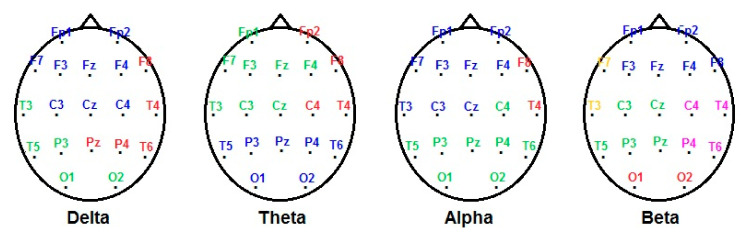
Topography of the factors resulting from the factor analysis of the z value of the electroencephalography (EEG) absolute power for each frequency band (delta, theta, alpha and beta). Factor 1 is represented in blue, factor 2 in green, factor 3 in red, factor 4 in pink, and factor 5 in yellow.

In order to reduce factors in EEG channels, factor analysis was performed with the main components’ method and a Varimax rotation, entering the z-scores for absolute power in each of the 19 electrodes for every band separately (i.e., delta, theta, alpha, and beta bands). Figure 2 shows the location of the resulting factors in each frequency band. In the delta frequency band, we obtained three factors: factor 1 (Fp1, Fp2, F3, F4, C3, C4, F7, Fz, Cz; 37.2% of variance explained), factor 2 (P3, 01, 02, T3, T5; 25.6%), and factor 3 (P4, F8, T4, T6, Pz; 25.6% of variance explained). In the theta frequency band: factor 1 (P3, P4, Pz, O1, O2, T5, T6; 30.6% of variance explained), factor 2 (Fp1, F3, F4, C3, F7, T3, Fz, Cz; 29.9% of variance explained), and factor 3 (Fp2, C4, F8, T4; 26.6% of variance explained). In the alpha frequency band: factor 1 (Fp1, Fp2, F3, F4, C3, F7, T3, Fz, Cz; 36.7% of variance explained), factor 2 (C4, P3, P4, O1, O2, T5, T6, Pz; 35.7% of variance explained), and factor 3 (F8, T4; 16.7% of variance explained). In the beta frequency band: factor 1 (Fp1, Fp2, F3, F4, F8, Fz; 27.1% of variance explained), factor 2 (C3, P3, T5, Cz, Pz; 23.2% of variance explained), factor 3 (O1, O2; 14% of variance explained), factor 4 (C4, P4, T4, T6; 13.8% of variance explained), and factor 5 (F7, T3; 8.2% of variance explained).

**Figure 3:**
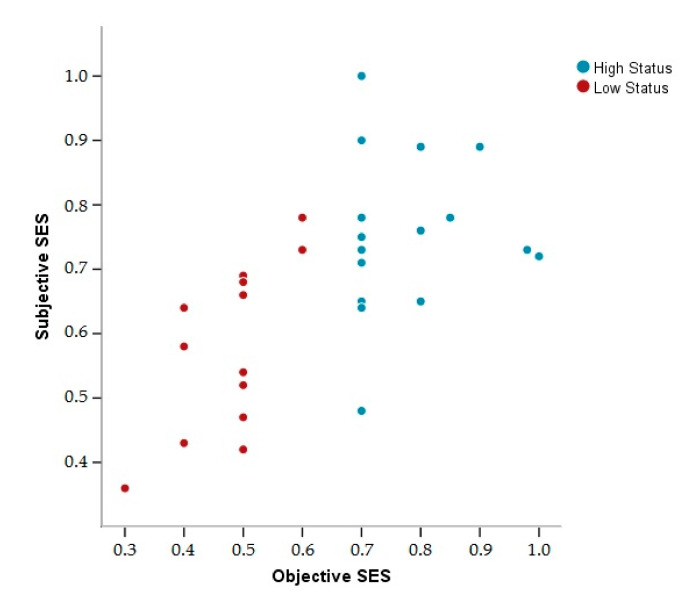
Scatterplot of the normalized objective and subjective socioeconomic status (SES) of the resulting groups.

In order to investigate the effect of objective and subjective socioeconomic status on hair cortisol concentration (HCC), EEG at resting condition, and cognitive performance, and considering that objective and subjective socioeconomic status are closely related (rho = 0. 653, *p* ≤ 0.0001), we created a new classification via a k-means cluster analysis of the entire sample (*n* = 30). The purpose of this cluster analysis was to sort out the sample into two groups, using the objective and subjective SES as the sorting criteria. Two clusters were identified: cluster 1 (*n* = 13) was characterized by low subjective and objective socioeconomic status (LS), and cluster 2 (*n* = 17) was characterized by high subjective and objective socioeconomic status (HS) (see Figure 3).

Non-parametrical analysis (U Mann–Whitney) was used to analyze differences between clusters on the demographic variables, hair cortisol concentration (HCC). Factors were created in each band of the EEG registration (at rest), and in cognitive indices of WAIS. We employed the clusters high socioeconomic status (HS) vs. low Socioeconomic status (LS) as between-subject factors.

All the *p*-values reported are two-tailed, and the level of significance was set at *p* = 0.05. When not otherwise specified, the results shown are means ± standard error of means (SEM). We used SPSS 22.0 to perform statistical analyses.

## 3. Results

### 3.1. Descriptive Data

The analyses revealed no main differences between clusters in age (mean ± SEM in HS: 67.78 ± 1.38; LS: 68.91 ± 1.41, *U* = 90.5, *p* = 0.41), or in intelligence quotient (mean ± SEM in HS: 121.35 ± 2.92; LS: 101.92 ± 7.29, *U* = 145.5, *p* = 0.142). Significant differences between clusters were found in objective socioeconomic status (AMAI), the HS cluster showed higher scores than the LS cluster (mean ± SEM in HS: 230 ± 6.4; LS: 159.92 ± 8.49, *U* = 221, *p* = 0.0001), and significant differences were also found between clusters in subjective socioeconomic status (SES) (mean ± SEM in HS: 7.3 ± 0.35; LS: 5.31 ± 0.41, *U* = 181.5, *p* = 0.031). Moreover, the HS group had significantly higher years of schooling than the LS group. (mean ± SEM in HS: 16.06 ± 0.69; LS: 13.85 ± 0.76, *U* = 162, *p* = 0.031).

### 3.2. Cognitive Performance

**Figure 4:**
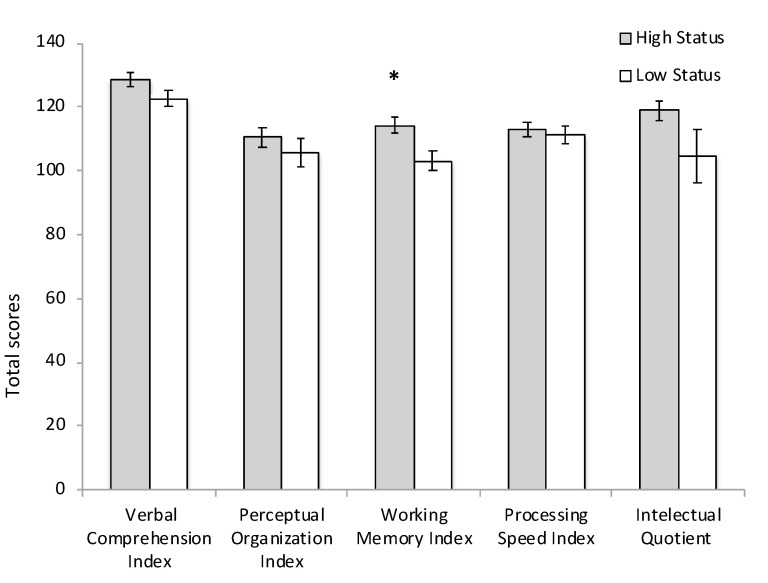
Total normalized scores by high and low status groups in each index and total IQ. * *p* = 0.009.

Those participants with higher status (HS group) scored higher in the Working Memory Index of WAIS (*p* = 0.009) and, as a trend, in total IQ (*p* = 0.08) (see Figure 4). Although the HS group scored higher in the other indices of WAIS, no other significant differences were found (all *p* ≥ 0.1).

### 3.3. EEG Measures

In Table 1, we can see how the HS cluster displayed lower z-values of AP in the delta (factor 3) and theta (factor 3) bands; these factors correspond with leads located at the frontotemporal and parietal leads in the right hemisphere for delta frequency band, and frontocentral and temporal leads also in the right hemisphere for the theta frequency band. In addition, in HS cluster, we observed higher alpha (frontotemporal leads) in the right hemisphere and beta frequencies in the left centrotemporoparietal leads (for more details about the location of each lead see Figure 2 and the Statistical Analysis Section).

### 3.4. Hair Cortisol Concentration (HCC)

The analysis revealed that the two clusters of socioeconomic status had similar concentrations of cortisol (mean ± SEM in HS: 24.74 ± 2.72, in LS: 23.72 ± 3.203, *U* = 75, *p* = 0.79).

## 4. Discussion

The present study focused on how objective and subjective SES influences psychophysiological and cognitive domains in healthy older people. To do this, EEG at rest with eyes closed was registered in order to explore the functional integrity of the nervous system, and we evaluated cognitive performance through WAIS subscales. We also acquired hair samples to obtain a measure of hair cortisol concentrations during the last three months as a chronic stress biomarker.

First, we differentiated between two groups; the first group is characterized by high SES, and the second group is characterized by low SES, understanding SES as a composite of subjective and objective measures.

Participants with higher SES had more years of schooling. The number of years of schooling constitutes one of the proxies most related to cognitive reserve [38]. Low education has been related to higher risk of development dementia and cognitive decline [39]. In addition, a higher SES also acts as a marker of cognitive reserve due to an environmental enrichment [40]. The cognitive reserve (CR) is a hypothetical construct formulated to explain the individual differences in cognitive performance of individuals who have had some neuropathological condition; in the face of a better cognitive reserve, the functional impairment of the patient is lower, that is, individuals with a better CR will compensate better for the effects of various factors involved in aging, including the gestation of a pathological process, via a more efficient use of the system [41].

### 4.1. SES Effects on Cognitive Performance

We expected that people with higher SES would show better cognitive performance. In particular, this occurred in the working memory domain. In this line, previous research has found that higher SES influences positively frontal white matter integrity [24] and higher SES has been related with better decision making, planning, and goal-directed behavior [42,43]. Although it is not yet clear which specific regions of the brain are involved in the working memory process, Christ et al. (2009) highlighted that the main specific regions involved in working memory include the anterior right prefrontal cortex, the right inferior parietal lobule, and the left middle frontal and precentral gyri [44].

However, no further differences in other cognitive domains were found. One possible explanation is that not all domains are affected by SES-related factors; several studies focused on the effect of SES on cognition during aging, which measured cognitive functions using MMSE [45,46]. One of these studies found that higher SES can act as a protector factor of age-related decline in cognitive function; the second study found that cognitive impairments in the elderly are independent of SES status. More recently, Zahodne et al. (2018) [30] found a positive association between lower SES and episodic memory; however, no effects of SSS on memory decline were found. With these latest findings in mind, we think that there is a necessity to analyze the various cognitive domains in a more structured manner during aging, and then to study cognitive processes in relation to SES and other psychosocial factors. Despite this, several signs point to the fact that education, SES, and SSS, may have a protective effect, but more studies with greater samples and controlling for confounder factors are needed.

### 4.2. SES Effects on EEG

Moreover, this group (high SES) also showed significantly lower delta power in frontotemporal leads and lower theta power in frontocentral and temporal leads of the right hemisphere. More delta and theta activity in a rest condition has been typically considered a sign of brain dysfunction. In patients with Alzheimer’s disease, specific alterations in the temporoparietal area of the brain right hemisphere have been described [47]. Moreover, Babiloni et al. (2016) suggest a direct relationship between resting-state cortical hypometabolism and synchronization of cortical neurons at delta rhythms in AD patients [48]; this was more evident in ventromedial frontal, associative temporoparietal, posterior cingulate, and precuneus areas. Furthermore, a decrease in amyloid β42 in cerebrospinal fluid significantly correlated with an increase in theta and delta activity [49].

In addition, the HS group also showed higher alpha frontotemporal and higher beta frequencies in the centrotemporoparietal leads. The activity in these frequency bands has been observed to be diminished in patients with MCI [48] and dementia [4,5], as compared with healthy elderly people; moreover, an increased *p*- and t-tau significantly correlated with decreased alpha and beta activity [49].

These findings suggest that regardless of their cognitive health indicators, such as good cognitive performance measured behaviorally, the group of low SES could have a higher probability of developing cognitive decline in the future. We concluded this based on previous research that has demonstrated that a slower EEG (i.e., more delta and theta power, as well as less alpha and beta activity) is a good predictor of future cognitive decline [2,3].

### 4.3. SES Effects on HCC

Although it seems that socioeconomic factors are important determinants of health and that they are closely related to psychosocial stress [50], previous findings that analyze the effects of these factors on the HPA axis function have yielded mixed results [51]. In addition, the implications of SES on HCC have been less studied. Studies focusing on children and adolescents have found higher HCC in families with low SES [52]. Other interesting results were found by O’ Brien et al. (2012) [53] in an adult population, where they revealed the importance of being part of a minority group with regard to HCC and chronic stress indicators, such as SES. Specifically, they found that people from minorities (e.g., Asian, Indian, Latino-Hispanic, etc.) with low and high SES showed the highest HCC, whereas people with mid-SES showed the lowest HCC. On the other hand, people from non-minorities with mid-SES showed the highest HCC, and those with highs SES showed decreases in HCC. In our case, our results failed to find significant differences in accumulated cortisol with regard to SES; one possible explanation, in addition to the above, could be that HCC might be reflecting other factors than psychosocial stress, considering that our sample consists of very healthy individuals, with medium-to-high status compared to the rest of the Mexican population. This typically involves having an active lifestyle, engaging in regular physical activity, and having more social interactions, which may trigger cortisol peaks during the day [54]. Hence, we think that the SES subjective perception could act as a potential stressor; however, the active lifestyle might serve as an offset, acting as a potential brain protector. Regardless, this is the first study to explore subjective and objective socioeconomic status effects on HCC in a healthy elderly population. Thus, future research might focus on the older population with different subjective social perceptions.

These results expand the knowledge about how subjective perceptions can influence health outcomes; however, we have to take these results with caution due to some limitations, such as the small sample size and the fact that the participants recruited are not representative of the Mexican population, so there might be some trouble regarding the generalization of the results. Despite these limitations, our results constitute the first evidence of the impact of SES on EEG activity and on cognitive function in healthy older people. Future research should further explore the relationship between psychosocial factors and successful aging, attending to the underlying biological processes.

## 5. Conclusions

The present study provides relevant information about social aspects such as the socioeconomic situation. Hence, these aspects have to be considered in the future of the aging process and in the prevention of cognitive decline.

We think that the main relevance of these results relies on the establishment of how psychosocial factors and, more importantly, subjective perceptions about an individual’s SES, are related to the development of age-related cognitive decline in people with similar affluence levels. With that in mind, the psychological work on subjective perceptions about oneself should be included in the social programs focused on successful aging.

## Figures and Tables

**Table 1:** Z Absolute power values of each factor separately by frequency bands.

Frequency Band	Factor	High Status (*n* = 17) (Mean ± SEM)	Low Status (*n* = 13) (Mean ± SEM)	U Mann–Whitney	*p*
Delta	Factor 1	0.100 ± 0.298	−0.165 ± 0.181	113	0.93
	Factor 2	−0.211 ± 0.245	0.098 ± 0.218	95	0.53
	Factor 3	−0.480 ± 0.225	0.602 ± 0.219	40	0.002
Theta	Factor 1	0.110 ± 0.237	−0.204 ± 0.293	131	0.41
	Factor 2	−0.021 ± 0.299	0.022 ± 0.189	100	0.68
	Factor 3	−0.304 ± 0.222	0.457 ± 0.274	61	0.039
Alpha	Factor 1	0.078 ± 0.198	−0.214 ± 0.326	114	0.9
	Factor 2	−0.006 ± 0.225	−0.011 ± 0.322	137	0.28
	Factor 3	0.225 ± 0.247	−0.395 ± 0.238	58	0.028
Beta	Factor 1	0.208 ± 0.211	−0.145 ± 0.301	150	0.10
	Factor 2	0.220 ± 0.214	−0.132 ± 0.283	139	0.24
	Factor 3	0.026 ± 0.248	−0.001 ± 0.289	142	0.19
	Factor 4	0.203 ± 0.231	−0.421 ± 0.232	88	0.36
	Factor 5	0.272 ± 0.262	−0.355 ± 0.237	164	0.025
